# Is Parenthood Contributing to Emotional Wellbeing? The Neutrality Paradox and a Possible Resolution

**DOI:** 10.1177/14747049261436325

**Published:** 2026-03-23

**Authors:** Menelaos Apostolou, Mark Sullman, Agata Błachnio, Ondrej Burysek, Ekaterina Bushina, Fran Calvo, William Costello, Tetiana Hill, Maria Galatiani Karageorgiou, Yanina Lisun, Denisse Manrique-Millones, Oscar Manrique-Pino, Yohsuke Ohtsubo, Aneta Przepiórka, Burcu Tekeş, Andrew Thomas, Yan Wang, Mads Larsen, Sílvia Font-Mayolas

**Affiliations:** 1 Department of Social Sciences, 121343 University of Nicosia, Nicosia, Cyprus; 2 Institute of Psychology, 49642 The John Paul II Catholic University of Lublin, Lublin, Poland; 3 College of Human and Health Sciences, 7759 Swansea University, Swansea, UK; 4 Center for Sociocultural Research, 68192 National Research University Higher School of Economics, Moscow, Russia; 5 Department of Pedagogy, Quality of Life Research Institute, 16738 Universitat de Girona, Catalonia, Spain; 6 Department of Psychology, 12330 University of Texas at Austin, The University of Texas at Austin, Austin, TX, USA; 7 Business School, 3769 University of Hertfordshire, Hatfield, UK; 8 Department of Journalism and Advertising, 187487 State University of Trade and Economics, Kyiv, Ukraine; 9 Department of Psychology, 187071 Universidad Científica del Sur, Lima, Peru; 10 251839 Universidad Católica Sedes Sapientiae, Lima, Perú; 11 Department of Social Psychology, Graduate School of Humanities and Sociology, 13143 University of Tokyo, Tokyo, Japan; 12 Department of Psychology, 589869 Başkent University, Ankara, Turkey; 13 Department of Psychology, 12478 Fudan University, Shanghai, China; 14 Department of Psychology, 8018 Norwegean University of Science and Technology, Trondheim, Norway; 15 Department of Psychology, Quality of Life Research Institute, 16738 Universitat de Girona, Girona, Spain

**Keywords:** parenthood, neutrality paradox, emotional wellbeing, life satisfaction, meaning in life

## Abstract

Evolutionary theorizing predicts that parenthood is associated with higher hedonic wellbeing (experiencing more positive and fewer negative emotions), higher eudaimonic wellbeing (experiencing greater meaning in life), and greater life satisfaction. To test this hypothesis, we analyzed a dataset comprising 5,556 participants drawn from 10 different nations. We found a small positive effect of parenthood on eudaimonic wellbeing, which was more pronounced for women. Conversely, we found virtually no differences between parents and nonparents across all measured dimensions of hedonic wellbeing and life satisfaction. Furthermore, for most dimensions, we did not detect significant interactions between parenthood status and sex, age, or relationship status. Additionally, participants with children reported lower relationship satisfaction than those without children; however, the observed difference was small. Our results contrast with evolutionary predictions, as well as empirical findings showing that parents perceive their children as sources of positive emotions and life purpose, creating a paradox for which we offer a possible resolution.

## Introduction

Having and raising children constitutes a central aspect of people's lives (Cowan & Cowan, 2000; Ko et al., 2020; Pick et al., 2022). Nevertheless, parenthood is a costly and demanding endeavor (Lino, 2014), raising questions regarding whether having children is associated with higher or lower emotional wellbeing, life satisfaction, and relationship satisfaction; questions that the present study aims to address. By the term “emotional wellbeing,” we refer to two distinguishable components. The first is hedonic wellbeing, which concerns the positive emotions (pleasant experiences such as happiness) and negative emotions (unpleasant experiences such as loneliness) that people commonly experience, as well as optimism. The second is eudaimonic wellbeing, which concerns one's sense of meaning and purpose in life (Ryan & Deci, 2001). Although these two components are correlated, they need not respond identically to the same life circumstances (Baumeister et al., 2013), and, as we argue below, evolutionary considerations generate distinct predictions for each. We will begin our discussion by presenting the evolutionary perspective on emotions.

### Emotional Wellbeing and Parenthood

Evolutionary theorizing proposes that emotions are adaptations designed to motivate fitness-enhancing behaviors, that is, behaviors that increase the probability that an individual's genetic material is represented in future generations (Nesse, 2019; Tooby & Cosmides, 2008). Specifically, negative emotions occur when individuals face situations that compromise fitness, prompting them to take corrective actions to alleviate these feelings. Conversely, positive emotions arise when individuals encounter situations that promote fitness, motivating them to maintain or seek similar situations in the future. For example, losing one's job can trigger sadness and anxiety, motivating people to seek new employment. On the other hand, receiving a job promotion may evoke feelings of pride and joy, motivating continued or enhanced performance at work to achieve further advancements. We need to say that not all emotions are grouped into positive and negative; but those that do not fall in such categorization (e.g., feeling curious) would have little impact on emotional wellbeing and life satisfaction that constitute the focus of the present study.

Children are the primary means by which people transfer their genetic material to future generations (Dawkins, 2016). Therefore, the main way for individuals to increase their fitness is to have children and raise them to sexual maturity (Stearns, 1992). This being the case, having and raising children should be associated with higher emotional wellbeing and life satisfaction. There are, however, instances in which having children could reduce fitness. For example, becoming pregnant without a supportive intimate partner or sufficient financial resources to raise a child can reduce fitness. If we make the reasonable assumption that, in contemporary societies, in most cases, having children reflects planning, where individuals have assessed that conditions are right to have children, then we can argue that having children is usually fitness-enhancing, and thus should be associated with higher emotional wellbeing and life satisfaction.

Moreover, because parents and children are closely genetically related, parental fitness is closely tied to the fitness of their children. Emotional mechanisms such as happiness are thought to track people's fitness and to be triggered accordingly (Nesse, 2019). It follows that positive emotions are elicited in parents when their children's fitness increases, and negative emotions when their children's fitness is compromised. In this way, people behave so as to increase their children's fitness, and thus their own, in order to experience positive emotions, and they act to protect their children from fitness-compromising circumstances, in order to avoid experiencing negative emotions. Consequently, children would be expected to serve as sources of both positive and negative emotions (Apostolou & Kagialis, 2025).

#### Hedonic and Eudaimonic Wellbeing

It is important to recognize that the evolutionary logic developed above does not predict a uniform effect across all components of emotional wellbeing. Hedonic and eudaimonic wellbeing are likely to be affected differently by parenthood, and evolutionary reasoning helps explain why. Regarding hedonic wellbeing, the fitness-tracking function of positive and negative affect implies that, *ceteris paribus*, parents would experience more negative as well as more positive emotions than nonparents. Given the positive contribution of children to fitness, we predict that the positive would be more than the negative emotions, so that parenthood has a positive effect on hedonic wellbeing.

Regarding eudaimonic wellbeing, the prediction is more straightforward. Having children represents a long-term, ongoing commitment to the most fitness-relevant project an organism can undertake: the production and rearing of offspring. From an evolutionary standpoint, mechanisms that sustain prolonged parental investment should be favored by selection (Trivers, 1972). A stable sense of meaning and purpose in life, rather than transient hedonic states, is well-suited to support such sustained commitment. In other words, the sense that one's life has direction and significance functions as a motivational scaffold that keeps parents engaged with the demanding, long-duration task of raising children, even when moment-to-moment affect is negative. Accordingly, the effect of parenthood should be stronger and more reliably detected for eudaimonic wellbeing (i.e., meaning in life) than for hedonic wellbeing (i.e., aggregate levels of positive or negative affect).

Overall, the foregoing arguments lead to the prediction that parenthood would be associated with increases in both hedonic and eudaimonic wellbeing, with the latter effect being more pronounced. Furthermore, to the extent that parenthood elevates overall emotional wellbeing, it should also be associated with higher life satisfaction, predicting that parents would report greater life satisfaction compared to nonparents. The existing empirical literature provides mixed support for these predictions.

### Current Literature

Hansen (2012), in a literature review addressing folk theories about parenthood, found that the belief that parenthood results in greater happiness appears pervasive worldwide. Recently, Apostolou and Kagialis (2025) employed a mixed-methods approach to ask Greek-speaking parents about possible advantages of parenthood. They identified seven distinct advantages, the most highly endorsed of which were “experience love and other positive emotions” (endorsed by over 97% of participants) and “give meaning to life” (endorsed by over 87%).

Aassve et al. (2012) used data from the European Social Survey—which included participants from 19 European nations—to examine the relationship between happiness and having children. Responding to the question, “How happy are you?” female participants showed a small positive association between happiness and parenthood, while no significant effect emerged for men. Similarly, Nelson et al. (2013) employed the World Values Survey dataset, which included a representative sample from the United States, measuring happiness, life satisfaction, and meaning in life. Initial analyses, without controlling for confounding variables, demonstrated that parents reported significantly higher scores on all three dimensions. After controlling for relationship status, however, only the measure of meaning in life retained significance, albeit with a small effect size.

Nelson-Coffey et al. (2019) analyzed data from the National Survey of Families and Households, a nationally representative U.S. dataset. Participants in this study responded to questions assessing happiness, depressive symptoms, and life satisfaction. Results indicated fathers reported more happiness and fewer depressive symptoms than men without children, whereas mothers reported similar depressive symptoms to non-mothers but less happiness. Yet, relationship status—a likely confounding variable—was not controlled. The authors also recruited another U.S. sample, measuring life satisfaction, positive emotions, and negative emotions, finding greater life satisfaction among fathers compared to men without children. Nevertheless, as in previous analyses, control for confounding variables was not included.

On the other hand, Stanca (2012) employed World Values Survey data obtained from multiple surveys conducted in 94 countries, measuring life satisfaction and happiness using single-item instruments. Findings revealed associations between parenthood and slightly lower happiness (a very small effect) and lower life satisfaction (a relatively small effect). Additional analyses suggested these negative effects were partially mediated by an adverse impact on financial satisfaction (see also Margolis & Myrskylä, 2011). A different study used data from the European Values Survey (EVS) covering the years 1981 to 2008 (Ugur, 2020). The survey included one question about happiness and another about life satisfaction, both of which the authors used as dependent variables. Controlling for various demographic factors, including relationship status, the study found that parenthood status had no statistically significant effect on happiness. Still, it had a positive and significant effect on life satisfaction: having children increased parents’ life satisfaction by 0.33 to 0.41 points on a 10-point scale. However, it was also found that for single parents having children was associated with negative effects on life satisfaction and happiness compared to nonparents.

### The Present Study

To date, three studies examining the association between parenthood and emotional wellbeing have identified some positive effects (i.e., Aassve et al., 2012; Nelson et al., 2013, 2019). A significant limitation within these studies, however, was a failure to adequately control relationship status as a confounding variable. Specifically, Aassve et al. (2012) and Nelson et al. (2013) employed relationship-status variables distinguishing only between married and single participants, with the latter group including both individuals who were not in any intimate relationship and those who were unmarried but in an intimate partnership, while Nelson-Coffey et al. (2019) did not control relationship status at all.

Adjusting for relationship status is crucial because individuals without intimate partners are significantly less likely to have children than those in partnerships. Extant research indicates that individuals in intimate relationships report higher levels of emotional wellbeing, life satisfaction, and purpose than unpartnered individuals (Apostolou et al., 2024; Apostolou & Kislev, 2026). Thus, when relationship status is uncontrolled, parenthood could inadvertently serve as a proxy for this variable, artificially inflating associations between parenthood and emotional wellbeing. Consequently, any observed positive effects in the aforementioned studies might reflect relationship status rather than true parenthood effects. Consistent with this argument, by controlling for relationship status, Ugur (2020) found no significant effect of parenthood status on happiness, and a small significant effect on life satisfaction. Yet, the study utilized only two single-item measures, limiting the scope and depth necessary to fully capture the varied dimensions of emotional wellbeing.

Furthermore, to our knowledge, only one study has reported negative effects of parenthood on emotional wellbeing (Stanca, 2012; replicated by Margolis & Myrskylä, 2011 using the same dataset). Nevertheless, the reported effects were extremely small and possibly only achieved significance due to the large sample size. This study also did not distinguish adequately between single individuals and those who were partnered but unmarried, while it utilized only two single-item measures for emotional wellbeing. Such measurement limitations have characterized most research in this domain, as available datasets were typically collected for broader survey purposes.

The present study addresses these limitations by examining the relationship between parenthood and multiple measures of emotional wellbeing, in addition to life satisfaction, while explicitly controlling for relationship status. Our primary hypothesis is that parenthood will be associated with higher hedonic and eudaimonic wellbeing and greater life satisfaction. As parenthood is associated with increased fitness in all cultural contexts, we predict further that these effects would be consistent across different cultural settings. Furthermore, previous research has revealed significant interactions involving parenthood status, sex, and relationship status (Aassve et al., 2012; Nelson et al., 2013), which our study also seeks to further investigate. Additionally, an important dimension connected to emotional wellbeing and life satisfaction is relationship satisfaction. Given existing empirical evidence identifying parenthood as negatively affecting relationship satisfaction (Don & Mickelson, 2014; Lawrence et al., 2008), the current study further seeks evidence to support this finding.

## Method

### Participants

In the current study, we analyzed the dataset from the Apostolou et al. (2024) study. The study employed a cross-cultural, cross-sectional design to investigate the association between relationship status and emotional wellbeing. The original study included participants from 12 countries: China, Egypt, Greece, Japan, Oman, Peru, Poland, Russia, Spain, Turkey, the United Kingdom, and Ukraine. However, for participants from Egypt and Oman, parenthood status was not recorded, so these samples were excluded from our analyses. Convenience sampling method was used so the samples are nonprobability (full details about the sampling method are reported in Apostolou et al., 2024). In effect, our analyzed sample comprised 5,556 participants (3,350 women, 2,189 men, and 17 participants who did not disclose their sex) (full demographic information is reported in the Supplemental material). The mean age was 33.1 years (*SD* = 13.8) for women and 36.0 years (*SD* = 15.0) for men. Furthermore, 37.5% of the participants identified as single, 31.6% as married, 25.8% as being in a relationship, and 5.1% described their relationship status as “other.” Additionally, 38.5% of participants reported having children. All data are available here: https://osf.io/94usy/?view_only=5b78d18f9ad74ddb9f727653874c1b93

### Materials

Life satisfaction was assessed using the Satisfaction with Life Scale (Diener et al., 1985). It involved a five-item instrument that asks participants to rate each item using a seven-point Likert scale (1—totally disagree, 7—totally agree) (Cronbach's alpha = 0.86).

Happiness was measured using the Happiness Measures (HM), comprising two separate items: The first required participants to rate their happiness on an 11-point scale (0 = very low, 10 = very high), while the second asked participants to estimate the proportion of time spent in happy, unhappy, and neutral moods (Fordyce, 1988). Positive and negative emotions were measured using the Positive and Negative Affect Schedule–Expanded Form (PANAS-X; Watson & Clark, 1999), specifically employing two basic negative emotion scales (Guilt—Cronbach's alpha = 0.87, Sadness—Cronbach's alpha = 0.89) and two basic positive emotion scales (Joviality—Cronbach's alpha = 0.93, Self-assurance—Cronbach's alpha = 0.90). To minimize respondent burden, the original study did not include broader positive and negative affect composites or assess additional emotions. Optimism was measured using a 10-item instrument (Cronbach's alpha = 0.79) developed by Scheier et al. (1994), where participants’ answers were recorded on a five-point scale (0—strongly disagree, 4—strongly agree).

Meaning in life was measured using a 10-item instrument (Cronbach's alpha = 0.76) developed by Steger et al. (2006), where answers were recorded in a seven-point Likert-type scale which ranged from 1 (absolutely true) to 7 (absolutely untrue). Relationship satisfaction was assessed with a seven-item scale (Cronbach's alpha = 0.92) developed by Hendrick (1988). Participants were asked to answer each item using a five-point scale ranging from 1 (low satisfaction) to 7 (high satisfaction). Finally, participants’ sex, age, parenthood status (whether they had children or not), and relationship status were recorded. Relationship status was classified into the following categories: “In a relationship,” “Married,” “Involuntarily single,” “Single between relationships,” “Prefer to be single,” and “Other.” The study was translated into the respective languages of each country in the sample using the back-translation method.

### Data Analysis

To address our research questions, we estimated a series of linear mixed models using the MIXED procedure in SPSS version 31. Participants (level 1) were nested within countries (level 2). For each of the 12 dependent variables, we specified a model with random intercepts for country and fixed effects for parenthood status, sex, relationship status, and age. Note that age effects were modeled as linear. Restricted Maximum Likelihood (REML) estimation was used. We also attempted to fit models with random slopes for parenthood to examine whether the parenthood effect varied across countries; however, these models did not converge due to the limited number of level-2 units, so we retained only the random-intercept specification. With respect to relationship satisfaction, the analysis was restricted to participants who indicated that they were in a relationship or married (N = 3,190). Specifically, we used a relationship status variable with two categories: “in a relationship” and “married.”

We performed a total of 12 multilevel tests. To reduce the probability of type I error associated with conducting multiple tests, we applied a Bonferroni correction, adjusting the alpha level to .004 (.05/12). This correction was applied to both fixed and interaction effects. We also examined our research questions individually for each sample. Specifically, we conducted a series of analysis of covariance (ANCOVA) tests where each dimension of interest was treated as the dependent variable. The categorical independent variables were sex, parenthood status, and relationship status; the continuous independent variable was age, which was modeled as linear. Individual subsample results are available in the Supplemental material.

## Results

As indicated by the raw scores in Table 1, participants who had children reported higher overall mean scores in happiness, meaning in life, optimism, and life satisfaction, as well as lower levels of unhappiness, guilt, and sadness compared to nonparents. Nevertheless, apart from the dimension of meaning in life, none of these differences were statistically significant for the pooled sample. Furthermore, the adjusted mean difference scores between parents and nonparents indicated a very high overlap between the two groups. For instance, for happiness measured on an 11-point scale, parents scored, on average, 0.32 units higher than nonparents. This can also be seen from the effect sizes of parenthood status for individual samples in Table 2, which in most cases were zero or near zero. In general, as indicated in Table 2 and detailed in the Supplemental material, there was cross-cultural consistency, with effect sizes ranging between zero and very small, and the main effects of parenthood being nonsignificant.

**Table 1.** The Effect of Parenthood Status on Emotional Wellbeing and Life Satisfaction.

	*Parenthood*				Parenthood * Sex	Parenthood * Age	Parenthood * Relationship Status
	*Yes*	*No*					
	Mean raw (SD)	Mean raw (SD)	Mean difference (CI: 95)	*p*-Value	*p*-Value	*p*-Value	*p*-Value
Happiness	6.81 (1.96)	6.18 (2.14)	0.32 (−0.33 0.97)	.657	1.00	.298	.671
Happy	51.17 (26.32)	44.54 (25.35)	2.40 (−5.51–10.31)	.954	.221	.174	.236
Unhappy	22.60 (20.59)	30.90 (23.49)	0.38 (−6.55–7.31)	.657	.478	.332	.885
Neutral	32.12 (23.01)	36.97 (23.34)	−2.49 (−9.94–4.96)	.285	.017	.910	.345
Joviality	3.14 (0.92)	3.13 (0.94)	0.18 (−0.10–0.45)	.206	.706	.931	.592
Self-assurance	3.03 (1.01)	3.05 (1.03)	0.40 (0.13–0.68)	.003	.002	.147	.072
Guilt	1.97 (0.87)	2.20 (0.95)	−0.08 (−0.37–0.22)	.637	.884	.285	.350
Sadness	2.11 (0.98)	2.50 (1.08)	−0.31 (−0.64–0.01)	.116	.404	.170	.213
Life satisfaction	4.41 (1.35)	4.20 (1.30)	.025 (−0.37–0.42)	.870	.730	.160	.609
Meaning in life	4.57 (1.01)	4.09 (1.02)	0.90 (0.58–1.22)	<.001	<.001	<.001	.115
Optimism	3.94 (0.72)	3.82 (0.73)	0.13 (−0.08–0.35)	.572	.356	.332	.653
Relationship satisfaction	4.36 (0.79)	4.64 (0.83)	−0.61 (−0.88 - −0.33)	<.001	.007	.198	.006

*Note.* The first two rows report the unadjusted means for parents and nonparents in the sample. The third row reports the mean difference between parents and nonparents, adjusted for the independent variables.

**Table 2.** Significant Main Effects and Effect Sizes of Parenthood Across Different Samples.

	China	Greece	Japan	Peru	Poland	Russia	Spain	Turkey	UK	Ukraine
	η_p_^2^	η_p_^2^	η_p_^2^	η_p_^2^	η_p_^2^	η_p_^2^	η_p_^2^	η_p_^2^	η_p_^2^	η_p_^2^
Happiness	.000	.003	.000	.001	.001	.001	.009	.010	.002	.000
Happy	.007	.001	.001	.000	.001	.001	.003	.003	.004	.002
Unhappy	.010	.003	.000	.000	.003	.003	.005	.001	.005	.000
Neutral	.001	.002	.002	.000	.005	.011	.017	.000	.001	.001
Joviality	.000	.007	.006	.001	.000	.003	.004	.005	.004	.000
Self-assurance	.000	.006	.000	.001	.001	.003	.002	.060	.001	.000
Guilt	.000	.005	.001	.000	.001	.005	.017	.000	.001	.003
Sadness	.000	.002	.000	.004	.000	.001	.001	.010	.003	.003
Life satisfaction	.002	.000	.003	.001	.000	.000	.015	.006	.003	.001
Meaning in life	.000	.020*	.002	.006	.000	.000	.008	.006	.001	.000
Optimism	.000	.005	.002	.001	.002	.004	.004	.006	.008	.007
Relationship satisfaction	.000	.028*	.010	.005	.005	.008	.008	.004	.017	.005

*p-Value ≤.004 (Bonferroni adjusted).

The observed differences in raw mean scores between parents and nonparents probably occurred because parents were more likely to be in intimate relationships, thereby experiencing more positive and fewer negative emotions compared to nonparents, who are more frequently single and thus experience fewer positive and more negative emotions. To test whether relationship status was confounding the results, we reanalyzed the data excluding relationship status as an independent variable. These analyses indicated significant parenthood effects across all examined dimensions; however, these effects disappeared when relationship status was included as an independent variable.

For the pooled sample, the association between parenthood and meaning in life was statistically significant, with parents reporting higher scores compared to nonparents. Still, the adjusted mean difference was relatively small, with parents scoring 0.90 units higher on a 10-point scale than nonparents. Furthermore, as presented in Table 2, the effect for meaning in life reached significance only for the Greek sample. For other samples, such as the Ukrainian sample, the effect size was zero. In addition, our analysis of the pooled sample detected no significant interactions between parenthood and sex, age, or relationship status for the majority of cases.

For self-assurance, a significant interaction between parenthood status and sex emerged. More specifically, when comparing the nonparent and parent groups, men's scores remained unaffected, while women's scores were higher. From the Supplemental material we can see that this interaction was not significant for most of the countries in the sample; still in many cases was close to the significance level. For meaning in life, a significant interaction between parenthood status and sex emerged. In particular, women's scores increased more than men's scores when comparing parents to nonparents. From the Supplemental material, we can see that this interaction was close to significance level for most countries in the sample. Moreover, a significant interaction with age also emerged. Here, scores were higher for the parent group compared to the nonparent group, and this increase was greater for older participants than for younger participants. From the Supplemental material we can see that for most countries this interaction was not significant.

Regarding relationship satisfaction in the pooled sample, there was a statistically significant main effect of parenthood, with participants who had children reporting lower satisfaction compared to participants without children. The difference was relatively small, with parents scoring 0.61 units lower on a seven-point scale. Table 2 further shows that the negative effect was statistically significant only for the Greek sample. Additionally, apart from China, the effect size of parenthood was above zero in other national samples, indicating that larger sample sizes might have resulted in significant main effects.

## Discussion

In the present study, we examined differences in emotional wellbeing, relationship satisfaction, and life satisfaction between individuals who had children and those who did not in a large sample of participants from 10 countries. Our findings indicated that parents and nonparents showed no significant differences across nearly all measured dimensions; this pattern held true both for the pooled sample and each individual national sample. In the pooled sample, parents reported significantly higher levels of meaning in life compared to nonparents, but the effect size was very small and significant only in the Greek sample. In most instances, there was a significant interaction between sex and parenthood status, with parenthood being associated with a greater increase in scores for women than for men. Participants who had children also reported lower relationship satisfaction than those without children, but again, this difference was small and significant only in the Greek sample.

Our results offer some support for the hypothesis that parenthood status is associated with increased eudaimonic wellbeing. Specifically, within the pooled sample, we found a significant fixed effect of parenthood status on meaning in life, with parents reporting higher scores than nonparents. However, this difference was small and statistically significant only for the Greek sample. Nevertheless, the effect approached significance in several other instances, suggesting that the difference might reach statistical significance in larger samples. Furthermore, a significant interaction emerged, indicating that the positive association between parenthood and meaning in life was more pronounced for women than for men. This interaction approached significance in most of the countries included in our sample. Overall, our findings suggest that parenthood is associated with a small increase in eudaimonic wellbeing, particularly for women.

These results do not support our hypothesis that parenthood is positively associated with hedonic wellbeing and life satisfaction. Instead, our findings suggest neutrality—namely, that parenthood has limited impact on these dimensions. We emphasize that in almost all cases, the observed differences between parents and nonparents were tiny, indicating that our lack of significant findings was unlikely attributable to insufficient statistical power. Although some variation existed among national samples, the conclusion that the effect of parenthood on hedonic wellbeing and life satisfaction was limited, was consistently supported across the studied countries.

We propose that previous research identifying a positive relationship between parenthood and hedonic wellbeing failed to effectively control for relationship status. In those studies, participants who were not in an intimate relationship—and thus less likely to have children—were often inadequately differentiated from participants who were unmarried but currently partnered—and thus more likely to have children. As a result, parenthood status served as a proxy for partnership status. Given that individuals in intimate partnerships typically report greater emotional wellbeing compared to single individuals, this methodological oversight likely inflated the positive effects attributed to parenthood. This argument is consistent with the findings of Ugur (2020), who controlled for relationship status and found no significant effect of parenthood on happiness. Additionally, previous research by Stanca (2012) revealed only a minuscule negative effect of parenthood on happiness, effectively indicating minimal practical impact regarding happiness. Yet, a more notable—although still small—negative effect of parenthood on life satisfaction was observed. On the other hand, Ugur (2020) found a significant positive effect of parenthood on life satisfaction, suggesting this dimension deserves further investigation.

In general, combining our findings with prior research suggests that parenthood is either neutral or has a very small impact on hedonic wellbeing and life satisfaction. This conclusion, however, creates a theoretical and empirical paradox. From a theoretical standpoint, parenthood greatly increases biological fitness, which evolutionary theorizing predicts would translate into parents experiencing positive emotions and elevated life satisfaction. Empirically, parents report experiencing their children as powerful sources of happiness and positive emotions. For example, Apostolou and Kagialis (2025) found that 97% of parents in their study strongly endorsed the view that their children constituted sources of positive emotional experiences. In the question “Watching children grow up is life's greatest joy” in the European Values Survey, about 90% of parents agreed (Ugur, 2020).

We propose a possible resolution to this paradox. To best increase their fitness, parents’ emotional mechanisms should monitor their children's fitness and correlate with it, but there is no need for lasting changes in parental wellbeing. Because parents and children are genetically related, and because children carry parents’ genetic material to future generations, an increase in children's fitness translates into an increase in parental fitness. Given this, positive emotions would be triggered to reward parents for achieving fitness-enhancing goals and to motivate them to invest in their children in order to experience these emotions again in the future.

For instance, graduating from a good university would raise children's fitness by making them more likely to secure good jobs and more attractive as mates, increasing the likelihood of finding a partner with high mate value. As parental fitness also increases, this event would trigger positive emotions such as joy, pride, and happiness. Experiencing these emotions would be one factor motivating parents to invest in their children's education. Conversely, dropping out of college would reduce children's fitness by lowering their prospects for good employment and for attracting high-quality mates. As parental fitness also declines, parents would experience negative emotions such as anger, disappointment, and sadness. Avoiding these emotions would be another factor motivating parents to invest in their children's education, as well as to advise them and influence them to focus on their studies.

In this respect, emotional mechanisms motivate fitness-increasing behavior. For this purpose, the emotional responses need not be lasting. For example, graduating from a good school with good marks would increase children's fitness and, by extension, their parents’ fitness. If parents became permanently happy and proud about this event, they might be less motivated to invest further in their children's college education to re-experience those emotions. Their fitness would be better served if they experienced a spike in positive emotions that returned to baseline after a while, thereby motivating continued investment to experience such spikes again. A similar pattern is observed among lottery winners, whose happiness initially spikes but eventually returns to baseline levels (Brickman et al., 1978).

Events that produce substantial changes in children's fitness, and thus strong parental emotional responses, such as graduating from college, are relatively infrequent, which makes their impact small in aggregate wellbeing measures. Nonetheless, parents remember them well. When, for example, parents are asked about the benefits of parenthood, memories of these positive emotions readily come to mind and shape their responses. This argument could potentially resolve the neutrality paradox: parenthood is associated with strong positive and negative emotions that parents recall and report, but these experiences are too infrequent and short-lived to affect aggregate measures. Our proposed resolution hinges on affective events tied to offspring milestones (e.g., graduating, dropping out) that would vary strongly with children's age, number of children, custodial status, and possibly children's sex, variable which were not measured in the present study. Thus, this resolution is not directly testable with the available variables, and needs to be examined by future research.

Additionally, positive emotions may frequently emerge from daily interactions with children, reinforcing the rewarding aspects of parenthood and motivating ongoing parental investment (Apostolou & Kagialis, 2025). Along these lines, Nelson et al. (2013) conducted a momentary emotional wellbeing study with 329 adults from the United States. Their participants reported daily emotional states through random pages over the course of seven days. They found parents consistently reported higher levels of momentary happiness, positive emotions, and meaning in life compared to nonparents. Using a day reconstruction method, these authors found that parents felt happier and experienced greater meaning when caring for their children compared to other daily activities. Nelson-Coffey et al. (2019) obtained similar results by contacting parents daily via a mobile application; parents reported significantly greater happiness during interactions with their children compared to noninteraction periods. Yet, daily interactions do not substantially increase fitness, causing these positive emotions to remain subtle and transient, thus making little difference in overall, aggregate emotional wellbeing. In relation to these findings, we need to emphasize that the observed “neutrality” of parenthood in our study refers to small between-group differences on aggregate measures, not absence of meaningful momentary experiences.

Evolutionary theorizing further proposes that parenthood would strengthen relationship bonds, as parents acquire high stakes in cooperating to raise offspring successfully. Supporting this, Apostolou and Kagialis (2025) found that parents perceived a relational advantage of parenthood for strengthening their partnerships. Correspondingly, empirical studies have demonstrated that children reduce the risk of divorce (Andersson, 1997). Yet, our current findings, along with prior research (Don & Mickelson, 2014; Lawrence et al., 2008), suggest a negative effect of parenthood on relationship satisfaction. Rather than fundamentally contradicting the evolutionary argument, perhaps a more accurate interpretation is that parenthood exerts two opposing influences: One positive, resulting from mutual genetic interests fostering cooperation and unity, and one negative, arising from associated financial costs, significant time demands, and stressors involved in child-rearing, all of which place strain on relationships.

We also need to recognize that the contemporary environment in which people have and raise children differs in many respects from the ancestral environment. For example, for most of our evolutionary history humans lived in close kin groups, whereas many people today lack access to nearby kin who can help raise offspring. As another example, in agropastoral, preindustrial societies older children often contributed economically through farm and herding labor, whereas in contemporary postindustrial societies children are typically financial liabilities because they require substantial monetary resources to raise. Because behavioral mechanisms, including emotions, evolved in a preindustrial context, they may not function optimally in the contemporary context to the extent that the two environments differ, a phenomenon known as the mismatch problem (Li et al., 2018). Nonetheless, we do not think the mismatch problem provides a solution to the neutrality paradox. For it to do so, novel environmental conditions would need to cause emotional mechanisms to fail to register fitness changes associated with having and raising children. We are not aware of such conditions, although this possibility warrants investigation in future research.

One concern that we would like to raise is that our findings could potentially discourage prospective parents (for the conditions people considered necessary for procreation see Apostolou et al., 2026). Given the substantial costs of parenting, individuals may conclude from limited impacts on hedonic wellbeing and potential negative effects on relationship satisfaction that choosing to remain childfree is preferable. We stress, however, that both our findings and related research remain insufficient to fully capture the depth and complexity of potential rewards and costs associated with parenthood. Therefore, these findings alone are not adequate for fully informed decision making regarding family planning. Nevertheless, our research does possess practical predictive value. Specifically, if one anticipates sustained increases in happiness and positive emotions from having children, these expectations will likely not materialize. Similarly, having children is unlikely to enhance relationship satisfaction, and instead may have neutral or slightly negative effects. In general, our results combined with the results of previous literature, suggest that parenting has many rewards, but a permanent increase in baseline hedonic wellbeing is unlikely to be one of them.

The study has several limitations. First, it relied on self-report data, susceptible to biases and inaccuracies inherent to subjective reporting. Second, our use of nonprobability samples limits the generalizability of our findings to broader populations. Additionally, while our study included parenthood status, we did not measure how the number of children might affect reported outcomes; future studies could address this issue. Moreover, we did not measure children's age or sex which could be associated with parents’ happiness (Pushkar et al., 2014). It could be the case that parents with young children have low emotional wellbeing and parents with older children have high emotional wellbeing, so in the aggregate the two effects cancel out. We do not think that this is the case, but future studies need to investigate this possibility.

Similarly, we did not measure related factors, such as financial status or level of education which could interact with parenthood and affect outcomes (Augustine & Negraia, 2024; Walsh & Murphy, 2021). Future research should replicate our findings while including a broader set of potentially important variables. Moreover, our sample included a broad set of postindustrial societies, but it did not include data from preindustrial societies. Future research should address this limitation by examining the association between emotional wellbeing and parenthood status in preindustrial contexts. Additionally, the study was not designed to formally test equivalence, and therefore the results should be interpreted as indicating limited observed differences rather than definitive evidence that no differences exist in the wellbeing between parents and nonparents.

In summary, although parenthood's overall impact on emotional wellbeing, relationship, and life satisfaction appears limited, these dimensions remain complex, multifactorial, and insufficiently understood. Existing evidence suggests a small positive effect on eudemonic wellbeing, and no permanent positive or negative effect on hedonic wellbeing and life satisfaction, and a possible small negative effect on relationship satisfaction. Additional, nuanced research is required for a comprehensive understanding of how having children affects emotional and relationship wellbeing.

## Supplemental Material

sj-docx-1-evp-10.1177_14747049261436325 - Supplemental material for Is Parenthood Contributing to Emotional Wellbeing? The Neutrality Paradox and a Possible Resolution Supplemental material, sj-docx-1-evp-10.1177_14747049261436325 for Is Parenthood Contributing to Emotional Wellbeing? The Neutrality Paradox and a Possible Resolution by Menelaos Apostolou, Mark Sullman, Agata Błachnio, Ondrej Burysek, Ekaterina Bushina, Fran Calvo, William Costello, Tetiana Hill, Maria Galatiani Karageorgiou, Yanina Lisun, Denisse Manrique-Millones, Oscar Manrique-Pino, Yohsuke Ohtsubo, Aneta Przepiórka, Burcu Tekeş, Andrew Thomas, Yan Wang, Mads Larsen and Sílvia Font-Mayolas in Evolutionary Psychology
