# Supplementary material for: Is Parenthood Contributing to Emotional Wellbeing? The Neutrality Paradox and a Possible Resolution
Source: Evol Psychol. 2026 Mar 23;24(1):14747049261436325. doi: 10.1177/14747049261436325 (PMC13009945; doi:10.1177/14747049261436325)
Supplement: sj-docx-1-evp-10.1177_14747049261436325 - Supplemental material for Is Parenthood Contributing to Emotional Wellbeing? The Neutrality Paradox and a Possible Resolution [file sj-docx-1-evp-10.1177_14747049261436325.docx]

**Supplementary Material**

The tables below present the results of the statistical analysis performed for each sample.

Table of contents

Demographics….2

China……… 3

Greece ……. 4

Japan……… 5

Peru……… 6

Poland…… 7

Russia…… 8

Spain……. 9

Turkey….. 10

UK……… 11

Ukraine… 12

Demographics

*Demographic information for the pooled and individual samples*

| **Countries** |  | *N* | *Age* | *Relationship status* | | | | | | *Parenthood* |
| --- | --- | --- | --- | --- | --- | --- | --- | --- | --- | --- |
|  |  |  | Mean (SD) | Single (difficulties in attracting mates) | Single (by choice) | Single (between relationships) | In a relationship | Married | Other | % Parents |
| Total |  | 5556 | 34.2 (14.3) | 12.2 | 19.3 | 9.1 | 23.5 | 29.4 | 6.5 | 38.5 |
| China |  | 427 | 27.8 (7.0) | 14.8 | 10.5 | 11.0 | 25.3 | 32.3 | 6.1 | 28.6 |
| Greece |  | 779 | 28.7 (11.1) | 17.6 | 13.0 | 13.0 | 28.3 | 20.8 | 7.2 | 25.4 |
| Japan |  | 622 | 50.2 (13.6) | 11.4 | 12.9 | 5.9 | 5.6 | 60.0 | 4.2 | 54.2 |
| Peru |  | 766 | 28.0 (10.5) | 6.4 | 32.9 | 11.0 | 28.5 | 15.1 | 6.1 | 29.9 |
| Poland |  | 558 | 46.5 (12.9) | 8.2 | 8.2 | 6.8 | 22.2 | 54.5 | 0.0 | 72.4 |
| Russia |  | 400 | 40.4 (11.1) | 9.3 | 7.8 | 7.5 | 15.5 | 56.3 | 3.8 | 72.3 |
| Spain |  | 410 | 36.1 (12.8) | 8.5 | 10.0 | 5.1 | 42.2 | 28.5 | 5.6 | 40.5 |
| Turkey |  | 733 | 30.2 (11.9) | 11.5 | 17.2 | 9.3 | 26.7 | 29.5 | 5.9 | 28.3 |
| UK |  | 461 | 34.6 (13.4) | 16.3 | 18.7 | 6.9 | 33.2 | 19.7 | 5.2 | 36.7 |
| Ukraine |  | 400 | 20.2 (6.4) | 16.8 | 19.3 | 18.8 | 36.6 | 3.3 | 5.3 | 3.5 |

China

|  |  | *Parenthood* | | | | *Parenthood * Sex* | | *Parenthood * Age* | | *Parenthood * Relationship status* | |
| --- | --- | --- | --- | --- | --- | --- | --- | --- | --- | --- | --- |
|  |  | *Yes* | *No* |  |  |  |  |  |  |  |  |
|  |  | Mean (SD) | Mean (SD) | *p*-value | η_p_^2^ | *p*-value | η_p_^2^ | *p*-value | η_p_^2^ | *p*-value | η_p_^2^ |
| Happiness |  | 7.79 (1.71) | 7.14 (1.70) | .732 | .000 | .771 | .000 | .785 | .000 | .046 | .023 |
| Happy |  | 52.50 (24.66) | 41.34 (21.32) | .097 | .007 | .649 | .001 | .199 | .004 | .161 | .016 |
| Unhappy |  | 21.97 (15.37) | 25.05 (14.45) | .049 | .010 | .129 | .006 | .071 | .008 | .099 | .019 |
| Neutral |  | 31.16 (20.91) | 35.27 (18.85) | .479 | .001 | .956 | .000 | .974 | .000 | .067 | .021 |
|  |  |  |  |  |  |  |  |  |  |  |  |
| Joviality |  | 3.17 (0.83) | 2.95 (0.84) | .897 | .000 | .828 | .000 | .845 | .000 | .524 | .008 |
| Self-assurance |  | 2.88 (0.91) | 2.56 (0.90) | .817 | .000 | .599 | .001 | .777 | .000 | .075 | .020 |
| Guilt |  | 1.88 (0.80) | 2.18 (0.99) | .698 | .000 | .417 | .002 | .672 | .000 | .111 | .018 |
| Sadness |  | 1.87 (0.85) | 2.21 (0.99) | .843 | .000 | .220 | .004 | .890 | .000 | .115 | .018 |
|  |  |  |  |  |  |  |  |  |  |  |  |
| Life satisfaction |  | 4.69 (1.23) | 4.13 (1.25) | .385 | .002 | .687 | .000 | .511 | .001 | .412 | .010 |
| Meaning in life |  | 4.30 (0.64) | 3.88 (0.80) | .892 | .000 | .631 | .001 | .558 | .001 | .668 | .006 |
| Optimism |  | 4.14 (0.53) | 3.94 (0.57) | .934 | .000 | .907 | .000 | .819 | .000 | .378 | .010 |
|  |  |  |  |  |  |  |  |  |  |  |  |
| Relationship satisfaction |  | 4.34 (0.65) | 4.47 (0.62) | .247 | .006 | .483 | .002 | .231 | .006 | .045 | .017 |

Greece

|  |  | *Parenthood* | | | | *Parenthood * Sex* | | *Parenthood * Age* | | *Parenthood * Relationship status* | |
| --- | --- | --- | --- | --- | --- | --- | --- | --- | --- | --- | --- |
|  |  | *Yes* | *No* |  |  |  |  |  |  |  |  |
|  |  | Mean (SD) | Mean (SD) | *p*-value | η_p_^2^ | *p*-value | η_p_^2^ | *p*-value | η_p_^2^ | *p*-value | η_p_^2^ |
| Happiness |  | 6.85 (2.02) | 6.55 (2.28) | .129 | .003 | .871 | .000 | .172 | .003 | .896 | .002 |
| Happy |  | 49.56 (25.71) | 48.29 (25.65) | .369 | .001 | .745 | .000 | .556 | .000 | .189 | .010 |
| Unhappy |  | 24.65 (21.49) | 28.45 (22.28) | .116 | .003 | .426 | .001 | .302 | .001 | .393 | .007 |
| Neutral |  | 35.37 (22.92) | 38.34 (22.24) | .285 | .002 | .729 | .000 | .677 | .000 | .789 | .003 |
|  |  |  |  |  |  |  |  |  |  |  |  |
| Joviality |  | 3.54 (0.83) | 3.40 (0.92) | .026 | .007 | .574 | .000 | .088 | .004 | .609 | .005 |
| Self-assurance |  | 3.34 (0.85) | 3.28 (0.91) | .040 | .006 | .218 | .002 | .097 | .004 | .059 | .014 |
| Guilt |  | 2.01 (0.90) | 2.11 (0.88) | .069 | .005 | .668 | .000 | .037 | .006 | .101 | .013 |
| Sadness |  | 2.59 (1.04) | 2.81 (1.06) | .191 | .002 | .707 | .000 | .376 | .001 | .253 | .009 |
|  |  |  |  |  |  |  |  |  |  |  |  |
| Life satisfaction |  | 4.71 (1.25) | 4.41 (1.27) | .729 | .000 | .405 | .001 | .720 | .000 | .847 | .003 |
| Meaning in life |  | 4.94 (1.13) | 4.16 (1.16) | <.001 | .020 | .664 | .000 | .023 | .007 | .180 | .010 |
| Optimism |  | 3.94 (0.70) | 3.77 (0.72) | .065 | .005 | .044 | .006 | .131 | .003 | .968 | .001 |
|  |  |  |  |  |  |  |  |  |  |  |  |
| Relationship satisfaction |  | 4.38 (0.96) | 4.83 (0.68) | .002 | .026 | .227 | .004 | .002 | .026 | .085 | .014 |

Japan

|  |  | *Parenthood* | | | | *Parenthood * Sex* | | *Parenthood * Age* | | *Parenthood * Relationship status* | |
| --- | --- | --- | --- | --- | --- | --- | --- | --- | --- | --- | --- |
|  |  | *Yes* | *No* |  |  |  |  |  |  |  |  |
|  |  | Mean (SD) | Mean (SD) | *p*-value | η_p_^2^ | *p*-value | η_p_^2^ | *p*-value | η_p_^2^ | *p*-value | η_p_^2^ |
| Happiness |  | 6.28 (1.89) | 5.25 (2.44) | .684 | .000 | .091 | .005 | .256 | .002 | .699 | .005 |
| Happy |  | 43.71 (28.02) | 34.24 (28.25) | .493 | .001 | .818 | .000 | .233 | .002 | .912 | .002 |
| Unhappy |  | 20.45 (21.50) | 27.79 (26.78) | .874 | .000 | .110 | .004 | .627 | .000 | .926 | .002 |
| Neutral |  | 35.84 (26.65) | 37.97 (29.13) | .409 | .002 | .251 | .002 | .437 | .001 | .813 | .004 |
|  |  |  |  |  |  |  |  |  |  |  |  |
| Joviality |  | 2.51 (0.79) | 2.31 (0.88) | .058 | .006 | .281 | .002 | .014 | .010 | .724 | .005 |
| Self-assurance |  | 2.05 (0.81) | 1.93 (0.87) | .654 | .000 | .076 | .005 | .714 | .000 | .614 | .006 |
| Guilt |  | 2.10 (0.87) | 2.34 (1.02) | .521 | .001 | .845 | .000 | .333 | .002 | .779 | .004 |
| Sadness |  | 2.14 (0.87) | 2.48 (1.06) | .776 | .000 | .537 | .001 | .681 | .000 | .950 | .002 |
|  |  |  |  |  |  |  |  |  |  |  |  |
| Life satisfaction |  | 3.77 (1.29) | 3.25 (1.35) | .151 | .003 | .080 | .050 | .031 | .008 | .595 | .006 |
| Meaning in life |  | 3.99 (0.73) | 3.78 (0.80) | .245 | .002 | .019 | .009 | .218 | .003 | .789 | .004 |
| Optimism |  | 4.08 (0.84) | 3.73 (0.99) | .316 | .002 | .019 | .009 | .499 | .001 | .560 | .006 |
|  |  |  |  |  |  |  |  |  |  |  |  |
| Relationship satisfaction |  | 4.57 (1.48) | 5.16 (1.33) | .075 | .008 | .099 | .007 | .177 | .005 | .675 | .002 |

Peru

|  |  | *Parenthood* | | | | *Parenthood * Sex* | | *Parenthood * Age* | | *Parenthood * Relationship status* | |
| --- | --- | --- | --- | --- | --- | --- | --- | --- | --- | --- | --- |
|  |  | *Yes* | *No* |  |  |  |  |  |  |  |  |
|  |  | Mean (SD) | Mean (SD) | *p*-value | η_p_^2^ | *p*-value | η_p_^2^ | *p*-value | η_p_^2^ | *p*-value | η_p_^2^ |
| Happiness |  | 7.25 (1.86) | 6.75 (2.07) | .439 | .001 | .163 | .003 | .397 | .001 | .095 | .012 |
| Happy |  | 60.20 (25.99) | 56.85 (24.98) | .979 | .000 | .357 | .001 | .640 | .000 | <.001 | .032 |
| Unhappy |  | 21.78 (23.13) | 30.25 (25.61) | .665 | .000 | .916 | .000 | .633 | .000 | .539 | .005 |
| Neutral |  | 34.34 (25.24) | 39.68 (25.41) | .890 | .000 | .256 | .002 | .668 | .000 | .733 | .004 |
|  |  |  |  |  |  |  |  |  |  |  |  |
| Joviality |  | 3.84 (0.78) | 3.65 (0.88) | .534 | .001 | .970 | .000 | .491 | .001 | .028 | .017 |
| Self-assurance |  | 3.93 (0.72) | 3.67 (0.88) | .316 | .001 | .015 | .008 | .623 | .000 | .061 | .014 |
| Guilt |  | 2.34 (1.03) | 2.56 (1.01) | .744 | .000 | .495 | .001 | .193 | .002 | .443 | .006 |
| Sadness |  | 2.28 (1.11) | 2.59 (1.10) | .071 | .004 | .536 | .001 | .018 | .007 | .169 | .010 |
|  |  |  |  |  |  |  |  |  |  |  |  |
| Life satisfaction |  | 5.17 (1.05) | 4.72 (1.23) | .315 | .001 | .487 | .001 | .155 | .003 | .074 | .013 |
| Meaning in life |  | 4.78 (0.97) | 4.34 (1.03) | .035 | .006 | .397 | .001 | .050 | .005 | .249 | .009 |
| Optimism |  | 4.27 (0.50) | 4.08 (0.57) | .322 | .001 | .328 | .001 | .479 | .001 | .716 | .004 |
|  |  |  |  |  |  |  |  |  |  |  |  |
| Relationship satisfaction |  | 4.39 (0.86) | 4.73 (0.57) | .532 | .001 | .761 | .000 | .119 | .007 | .028 | .022 |

Poland

|  |  | *Parenthood* | | | | *Parenthood * Sex* | | *Parenthood * Age* | | *Parenthood * Relationship status* | |
| --- | --- | --- | --- | --- | --- | --- | --- | --- | --- | --- | --- |
|  |  | *Yes* | *No* |  |  |  |  |  |  |  |  |
|  |  | Mean (SD) | Mean (SD) | *p*-value | η_p_^2^ | *p*-value | η_p_^2^ | *p*-value | η_p_^2^ | *p*-value | η_p_^2^ |
| Happiness |  | 6.58 (2.12) | 6.20 (1.95) | .451 | .001 | .133 | .004 | .451 | .001 | .640 | .005 |
| Happy |  | 51.32 (26.76) | 45.19 (26.27) | .565 | .001 | .869 | .000 | .811 | .000 | .884 | .002 |
| Unhappy |  | 20.18 (19.09) | 23.54 (19.35) | .239 | .003 | .218 | .003 | .433 | .001 | .367 | .008 |
| Neutral |  | 28.03 (22.48) | 31.27 (22.84) | .087 | .005 | .223 | .003 | .345 | .002 | .374 | .008 |
|  |  |  |  |  |  |  |  |  |  |  |  |
| Joviality |  | 3.14 (0.87) | 3.05 (0.89) | .962 | .000 | .647 | .000 | .964 | .000 | .765 | .003 |
| Self-assurance |  | 2.97 (0.80) | 2.82 (0.90) | .410 | .001 | .690 | .000 | .438 | .001 | .751 | .004 |
| Guilt |  | 1.85 (0.81) | 1.82 (0.79) | .404 | .001 | .792 | .000 | .949 | .000 | .657 | .004 |
| Sadness |  | 2.02 (0.96) | 2.08 (0.98) | .820 | .000 | .187 | .003 | .619 | .000 | .886 | .002 |
|  |  |  |  |  |  |  |  |  |  |  |  |
| Life satisfaction |  | 4.34 (1.33) | 4.07 (1.31) | .737 | .000 | .542 | .001 | .573 | .001 | .871 | .002 |
| Meaning in life |  | 4.47 (1.00) | 4.11 (0.99) | .924 | .000 | .142 | .004 | .754 | .000 | .969 | .001 |
| Optimism |  | 3.97 (0.53) | 3.87 (0.63) | .342 | .002 | .891 | .000 | .438 | .001 | .766 | .003 |
|  |  |  |  |  |  |  |  |  |  |  |  |
| Relationship satisfaction |  | 4.25 (0.94) | 4.45 (0.88) | .568 | .001 | .381 | .002 | .311 | .002 | .916 | .000 |

Russia

|  |  | *Parenthood* | | | | *Parenthood * Sex* | | *Parenthood * Age* | | *Parenthood * Relationship status* | |
| --- | --- | --- | --- | --- | --- | --- | --- | --- | --- | --- | --- |
|  |  | *Yes* | *No* |  |  |  |  |  |  |  |  |
|  |  | Mean (SD) | Mean (SD) | *p*-value | η_p_^2^ | *p*-value | η_p_^2^ | *p*-value | η_p_^2^ | *p*-value | η_p_^2^ |
| Happiness |  | 6.63 (2.03) | 6.39 (2.20) | .636 | .001 | .073 | .008 | .657 | .001 | .041 | .030 |
| Happy |  | 45.60 (27.21) | 42.24 (26.49) | .570 | .001 | .056 | .010 | .504 | .001 | .125 | .022 |
| Unhappy |  | 26.04 (22.64) | 30.04 (24.85) | .256 | .003 | .554 | .001 | .191 | .005 | .356 | .014 |
| Neutral |  | 34.44 (22.41) | 35.36 (22.08) | .045 | .011 | .397 | .002 | .005 | .020 | .221 | .018 |
|  |  |  |  |  |  |  |  |  |  |  |  |
| Joviality |  | 2.70 (0.73) | 2.73 (0.75) | .279 | .003 | .244 | .004 | .159 | .005 | .315 | .015 |
| Self-assurance |  | 2.69 (0.84) | 2.70 (0.85) | .272 | .003 | .402 | .002 | .457 | .001 | .111 | .023 |
| Guilt |  | 1.71 (0.73) | 1.94 (0.98) | .181 | .005 | .262 | .003 | .377 | .002 | .093 | .024 |
| Sadness |  | 2.02 (0.97) | 2.23 (1.10) | .517 | .001 | .539 | .001 | .600 | .001 | .137 | .022 |
|  |  |  |  |  |  |  |  |  |  |  |  |
| Life satisfaction |  | 3.56 (1.25) | 3.39 (1.24) | .846 | .000 | .211 | .004 | .540 | .001 | .093 | .024 |
| Meaning in life |  | 4.65 (0.98) | 4.23 (1.10) | .804 | .000 | .005 | .021 | .214 | .004 | .285 | .016 |
| Optimism |  | 3.40 (0.55) | 3.29 (0.61) | .232 | .004 | .441 | .002 | .370 | .002 | .057 | .028 |
|  |  |  |  |  |  |  |  |  |  |  |  |
| Relationship satisfaction |  | 2.88 (0.50) | 2.97 (0.40) | .014 | .021 | .688 | .001 | .052 | .014 | .003 | .040 |

Spain

|  |  | *Parenthood* | | | | *Parenthood * Sex* | | *Parenthood * Age* | | *Parenthood * Relationship status* | |
| --- | --- | --- | --- | --- | --- | --- | --- | --- | --- | --- | --- |
|  |  | *Yes* | *No* |  |  |  |  |  |  |  |  |
|  |  | Mean (SD) | Mean (SD) | *p*-value | η_p_^2^ | *p*-value | η_p_^2^ | *p*-value | η_p_^2^ | *p*-value | η_p_^2^ |
| Happiness |  | 7.38 (1.50) | 7.05 (1.58) | .056 | .009 | .761 | .000 | .026 | .013 | .619 | .007 |
| Happy |  | 58.81 (22.89) | 53.05 (20.87) | .329 | .003 | .221 | .004 | .477 | .001 | .301 | .013 |
| Unhappy |  | 17.54 (17.26) | 21.01 (16.46) | .150 | .005 | .813 | .000 | .144 | .006 | .148 | .016 |
| Neutral |  | 30.05 (20.19) | 30.09 (17.79) | .011 | .017 | .056 | .010 | .043 | .011 | .614 | .007 |
|  |  |  |  |  |  |  |  |  |  |  |  |
| Joviality |  | 3.50 (0.69) | 3.35 (0.76) | .194 | .004 | .895 | .000 | .036 | .011 | .730 | .005 |
| Self-assurance |  | 3.53 (0.75) | 3.33 (0.79) | .441 | .002 | .669 | .000 | .157 | .005 | .899 | .003 |
| Guilt |  | 2.39 (0.75) | 2.57 (0.81) | .010 | .017 | .261 | .003 | .013 | .016 | .208 | .015 |
| Sadness |  | 1.81 (0.86) | 2.15 (0.98) | .536 | .001 | .677 | .000 | .171 | .005 | .624 | .007 |
|  |  |  |  |  |  |  |  |  |  |  |  |
| Life satisfaction |  | 5.21 (0.98) | 4.83 (1.02) | .017 | .015 | .391 | .002 | .002 | .024 | .561 | .008 |
| Meaning in life |  | 4.91 (0.96) | 4.19 (0.96) | .088 | .008 | .045 | .010 | .368 | .002 | .543 | .008 |
| Optimism |  | 4.32 (0.67) | 4.04 (0.66) | .196 | .004 | .964 | .000 | .224 | .004 | .351 | .012 |
|  |  |  |  |  |  |  |  |  |  |  |  |
| Relationship satisfaction |  | 4.52 (0.67) | 4.87 (0.58) | .108 | .009 | .821 | .000 | .382 | .003 | .518 | .005 |

Turkey

|  |  | *Parenthood* | | | | *Parenthood * Sex* | | *Parenthood * Age* | | *Parenthood * Relationship status* | |
| --- | --- | --- | --- | --- | --- | --- | --- | --- | --- | --- | --- |
|  |  | *Yes* | *No* |  |  |  |  |  |  |  |  |
|  |  | Mean (SD) | Mean (SD) | *p*-value | η_p_^2^ | *p*-value | η_p_^2^ | *p*-value | η_p_^2^ | *p*-value | η_p_^2^ |
| Happiness |  | 7.12 (1.56) | 6.10 (2.24) | .007 | .010 | .218 | .002 | .183 | .003 | .380 | .008 |
| Happy |  | 61.13 (20.01) | 48.22 (22.57) | .127 | .003 | .536 | .001 | .784 | .000 | .126 | .012 |
| Unhappy |  | 28.02 (19.10) | 34.74 (21.78) | .356 | .001 | .332 | .001 | .290 | .002 | .004 | .024 |
| Neutral |  | 27.41 (19.92) | 31.58 (23.37) | .870 | .000 | .172 | .003 | .571 | .000 | .003 | .026 |
|  |  |  |  |  |  |  |  |  |  |  |  |
| Joviality |  | 3.45 (0.85) | 3.17 (0.90) | .062 | .005 | .098 | .004 | .423 | .001 | .326 | .008 |
| Self-assurance |  | 3.91 (0.75) | 3.51 (1.00) | .040 | .060 | .023 | .007 | .527 | .001 | .517 | .006 |
| Guilt |  | 1.72 (0.72) | 2.11 (0.96) | .862 | .000 | .680 | .000 | .957 | .000 | .816 | .003 |
| Sadness |  | 2.08 (0.94) | 2.70 (1.12) | .008 | .010 | .823 | .000 | .072 | .005 | .219 | .010 |
|  |  |  |  |  |  |  |  |  |  |  |  |
| Life satisfaction |  | 4.78 (1.18) | 4.41 (1.27) | .035 | .006 | .465 | .001 | .376 | .001 | .352 | .008 |
| Meaning in life |  | 5.26 (1.06) | 4.01 (1.02) | .049 | .006 | .006 | .011 | .620 | .000 | .308 | .009 |
| Optimism |  | 4.08 (0.71) | 3.69 (0.74) | .037 | .006 | .906 | .000 | .485 | .001 | .192 | .011 |
|  |  |  |  |  |  |  |  |  |  |  |  |
| Relationship satisfaction |  | 4.60 (0.71) | 4.73 (0.69) | .452 | .001 | .630 | .001 | .829 | .000 | <.001 | .031 |

UK

|  |  | *Parenthood* | | | | *Parenthood * Sex* | | *Parenthood * Age* | | *Parenthood * Relationship status* | |
| --- | --- | --- | --- | --- | --- | --- | --- | --- | --- | --- | --- |
|  |  | *Yes* | *No* |  |  |  |  |  |  |  |  |
|  |  | Mean (SD) | Mean (SD) | *p*-value | η_p_^2^ | *p*-value | η_p_^2^ | *p*-value | η_p_^2^ | *p*-value | η_p_^2^ |
| Happiness |  | 6.60 (1.99) | 6.09 (2.24) | .404 | .002 | .859 | .000 | .306 | .002 | .235 | .015 |
| Happy |  | 46.24 (23.52) | 35.71 (21.27) | .207 | .004 | .928 | .000 | .049 | .009 | .811 | .005 |
| Unhappy |  | 22.96 (18.97) | 25.08 (19.86) | .148 | .005 | .682 | .000 | .331 | .002 | .464 | .010 |
| Neutral |  | 32.28 (19.71) | 38.77 (20.40) | .539 | .001 | .899 | .000 | .844 | .000 | .539 | .009 |
|  |  |  |  |  |  |  |  |  |  |  |  |
| Joviality |  | 3.06 (0.93) | 3.04 (0.92) | .181 | .004 | .633 | .001 | .235 | .003 | .693 | .007 |
| Self-assurance |  | 2.77 (0.84) | 2.65 (0.90) | .549 | .001 | .328 | .002 | .333 | .002 | .931 | .003 |
| Guilt |  | 1.92 (0.93) | 2.17 (0.98) | .542 | .001 | .567 | .001 | .735 | .000 | .632 | .008 |
| Sadness |  | 2.13 (1.03) | 2.46 (1.05) | .234 | .003 | .812 | .000 | .360 | .002 | .316 | .013 |
|  |  |  |  |  |  |  |  |  |  |  |  |
| Life satisfaction |  | 4.61 (1.29) | 4.20 (1.34) | .234 | .003 | .959 | .000 | .147 | .005 | .492 | .010 |
| Meaning in life |  | 4.15 (0.83) | 4.00 (0.96) | .502 | .001 | .907 | .000 | .907 | .000 | .971 | .002 |
| Optimism |  | 3.38 (0.77) | 3.17 (0.66) | .061 | .008 | .355 | .002 | .090 | .006 | .049 | .025 |
|  |  |  |  |  |  |  |  |  |  |  |  |
| Relationship satisfaction |  | 3.44 (0.45) | 3.51 (0.39) | .033 | .019 | .009 | .028 | .019 | .023 | .305 | .010 |

Ukraine

|  |  | *Parenthood* | | | | *Parenthood * Sex* | | *Parenthood * Age* | | *Parenthood * Relationship status* | |
| --- | --- | --- | --- | --- | --- | --- | --- | --- | --- | --- | --- |
|  |  | *Yes* | *No* |  |  |  |  |  |  |  |  |
|  |  | Mean (SD) | Mean (SD) | *p*-value | η_p_^2^ | *p*-value | η_p_^2^ | *p*-value | η_p_^2^ | *p*-value | η_p_^2^ |
| Happiness |  | 4.57 (2.62) | 4.34 (1.81) | .771 | .000 | .590 | .001 | .504 | .001 | .091 | .021 |
| Happy |  | 30.57 (18.00) | 28.93 (20.54) | .414 | .002 | .092 | .007 | .364 | .002 | .968 | .001 |
| Unhappy |  | 40.14 (18.78) | 50.89 (22.92) | .854 | .000 | .460 | .001 | .776 | .000 | .288 | .013 |
| Neutral |  | 31.14 (15.63) | 44.57 (22.77) | .652 | .001 | .548 | .001 | .670 | .000 | .837 | .004 |
|  |  |  |  |  |  |  |  |  |  |  |  |
| Joviality |  | 2.21 (0.71) | 2.78 (0.70) | .829 | .000 | .072 | .008 | .906 | .000 | .573 | .008 |
| Self-assurance |  | 2.39 (0.85) | 2.77 (0.82) | .788 | .000 | .019 | .014 | .995 | .000 | .927 | .002 |
| Guilt |  | 1.68 (1.06) | 1.87 (0.71) | .247 | .003 | .246 | .003 | .272 | .003 | <.001 | .073 |
| Sadness |  | 2.21 (1.03) | 2.37 (0.98) | .315 | .003 | .639 | .001 | .475 | .001 | .015 | .032 |
|  |  |  |  |  |  |  |  |  |  |  |  |
| Life satisfaction |  | 3.50 (1.31) | 3.98 (0.99) | .504 | .001 | .584 | .001 | .425 | .002 | .333 | .012 |
| Meaning in life |  | 4.31 (0.94) | 4.10 (1.05) | .911 | .000 | .010 | .017 | .422 | .002 | .768 | .005 |
| Optimism |  | 3.95 (0.50) | 4.15 (0.51) | .102 | .007 | .007 | .019 | .047 | .010 | .572 | .008 |
|  |  |  |  |  |  |  |  |  |  |  |  |
| Relationship satisfaction |  | 3.95 (1.05) | 4.78 (0.68) | .616 | .002 | .469 | .003 | .565 | .002 | .478 | .002 |
